# Aging and distractor resistance in working memory: Does emotional valence matter?

**DOI:** 10.1186/s40359-022-00953-y

**Published:** 2022-11-04

**Authors:** Lin-jie Ding, Shao-shuai Zhang, Ming Peng, Xu Li

**Affiliations:** 1 Key Laboratory of Adolescent Cyberpsychology and Behavior (CCNU), Ministry of Education, Wuhan, China; 2 School of Psychology, Central China Normal University, No. 382, XiongChu Road, Hongshan District, Wuhan, 430079 Hubei Province China

**Keywords:** Working memory, Emotional distractor, Older adults, Young adults, Event-related potential, P2

## Abstract

**Background:**

Emotional stimuli used as targets of working memory (WM) tasks can moderate age-related differences in WM performance, showing that aging is associated with reductions in negativity bias. This phenomenon is referred to as the positivity effect. However, there is little research on whether emotional distractors have a similar moderating effect. Moreover, the underlying neural mechanism of this effect has not been studied. In this study, we examined the behavioral and neurophysiological basis for age differences in resistance to emotional distractors within WM.

**Methods:**

Older adults (*n* = 30, ages 60–74) and young adults (*n* = 35, ages 19–26) performed a 2-back task in which a digit was superimposed on a face with a happy, angry, or neutral expression as a distractor. Event-related potential (ERP) was simultaneously recorded to assess P2, N2, and later positive potential (LPP) amplitudes.

**Results:**

Older adults were less accurate and slower than young adults on the WM task. Moreover, the results demonstrated a significant interaction between age and emotional valence on response accuracy, young adults' performance was worse when the distractor was neutral or positive than when it was negative, but there was no effect of the emotional valence of distractors on older adults’ WM performance. ERP analyses revealed greater P2 amplitude in older adults than young adults, regardless of the emotional valence of distractors. However, older adults and young adults did not differ on N2 or LPP amplitude, and negative distractors elicited greater N2 than positive distractors in both age groups.

**Conclusions:**

The behavioral findings provided evidence of age-related reductions in negativity bias. Thus, the behavioral measures indicated a positivity effect in WM. However, the ERP results did not show this same interaction. These discrepant results raise questions about whether and to what extent older and young adults differ in controlling the effect of emotional distractors in WM.

**Supplementary Information:**

The online version contains supplementary material available at 10.1186/s40359-022-00953-y.

## Background

Aging is related to substantial cognitive declines. According to the inhibitory deficit hypothesis, deficient inhibitory processes are a major cause of these declines in older adults [1]. Older adults have been shown to be more vulnerable to distractors than young adults across a variety of paradigms [2, 3]. This common finding highlights the importance of clarifying the specific processes involved in older adults' relatively poorer ability to resist interference from distractors. Notably, working memory (WM) is among the cognitive domains that are most affected by inhibitory deficits, and failure in distractor resistance helps explain age-related reduction in WM capacity [4, 5]. However, this line of research has rarely considered the emotional valence of the distractors, a topic that is potentially important given the growing literature regarding emotion-cognition interactions and their relevance to aging.

A common idea in the literature on age-related changes in the processing of emotional information is that there is a positivity effect with aging. This term refers to age-related shifts away from negative information or towards positive information during cognitive processing [6, 7]. There has been an accumulation of empirical evidence for age-related positivity effect across various cognitive domains, such as attention [8], working memory [9], and episodic memory [10]. The positivity effect is often discussed within the theoretical framework of the socioemotional selectivity theory (SST) [11]. Several alternative theoretical models have also emerged to account for this phenomenon. These models differ in their predictions regarding the mechanism by which aging impacts emotional processing in cognition [12, 13] and are referred to as second generation SST models.

Three of these second-generation models have had the most influence in this literature. The cognitive control hypothesis highlights the potential importance of top-down cognitive control in achieving task goals. According to this hypothesis, elderly people selectively exert cognitive control resources to pursue their emotional goals [14]. The aging brain model posits that the reduced responses to negative stimuli in older adults result from decreases in brain activity in some regions, such as the amygdala, that are responsible for emotional processing [15]. Finally, the dynamic integration theory argues that the positivity effect is a byproduct of cognitive aging [16]. Older adults have difficulties in managing cognitive-affective complexity and they prioritize positive information via an automatic process rather than through controlled resource allocation, as would be suggested by the cognitive control hypothesis.

There have been inconsistent results regarding the positivity effect in WM, and some studies have failed to detect this phenomenon [17, 18]. Moreover, the great majority of studies on WM have focused on age differences in processing goal-relevant emotional stimuli [9, 19, 20]. It remains unclear whether the valence of emotional distractors affect WM performance in older and young adults differently. Thus, although much effort has been devoted to investigating the age-related positivity effect in WM, there is still debate about its underlying mechanisms [13, 21].

Only a few studies have tested older adults' performance in resisting emotional distractors in WM tasks, and the results have been mixed. In a study on visual short-term memory, older adults were less accurate in remembering the position of negative pictures than neutral pictures, whereas young adults' performance did not differ across these two valence conditions [22]. In another study, older adults were slower in resisting negative than neutral distractors in a 2-back task [23]. However, Oren et al. [24] found no differences between older and young adults in resisting negative or neutral information. Furthermore, other research suggested that older adults' WM performance was more negatively affected by positive and negative distractors than by neutral distractors, whereas no difference across these conditions was observed in young adults [25]. Unfortunately, however, very few studies [18, 25] simultaneously examined the effect of negative and positive emotional distractors on WM function in older adults within one study. In addition, no studies to our knowledge have examined the neurophysiological effect of negative and positive emotional distractors on WM function in older adults. The current study aims to fill this gap in the literature by using the event-related potential (ERP) technique to assess the neurophysiological responses during an emotional 2-back task in older and young adults.

Research using ERP measures has demonstrated some evidence of age differences in the time course of WM processing. Differences were most evident on the P2, N2 and P3 components. For example, some studies found that older adults had smaller N2 amplitudes, and larger P2 and P3 amplitudes, than young adults during WM tasks [26, 27]. However, smaller P2 [28] and smaller P3 amplitudes [29, 30] in older adults were also reported. The frontal P2 component reflects early WM processing, which involves the top-down comparison between sensory inputs and memory representations. Prior studies using the *n*-back paradigm have provided robust empirical evidence of the role of P2 in estimating individual differences in WM [31, 32]. N2, which is usually maximal in the frontal or fronto-central regions at the midline, relates to stimulus discrimination and conflict monitoring [33], with conflict trials resulting in greater N2 amplitude than non-conflict trials. Additionally, N2 increases with WM load, as more effort is required due to higher task demands [34]. Older adults were reported in one study to have smaller N2 than young adults, indicating decreased WM processing with aging [26]. The parietal P3 (also called P300) component indicates response selection and maintenance of the late updating process in WM. In another study using a WM task involving emotional materials, there was evidence of the later positive potential (LPP) component, a sustained positivity at midline and parietal sites, rather than the P3 component [31]. While some studies with young adults showed that negative and positive stimuli elicited larger LPP amplitude than neutral stimuli [35, 36], others failed to find an effect of emotional valence on P2 or LPP amplitude [31, 37]. It is still unclear how emotional distractors impact WM-related neural processing in older adults. Studies using the change-detection task to assess visual WM provide preliminary evidence of older adults' deficits in resisting emotionally neutral distractors, and older adults, compared to young adults, show a deficit in early filtering process in this task [5, 38]. Thus, there is a lack of studies on the neurophysiological effect of emotional distractors on WM processing among older adults.

In the present study we investigated age differences in the ability to resist interference from emotional distractors during a 2-back task. The *n*-back task is the most commonly used paradigm in investigations of aging-related changes in WM functioning [39]. Participants were instructed to compare a digit at trial *n* to that at trial *n* − 2. The digit was superimposed on a face with a happy, angry or neutral facial expression. The emotional distractors appeared in the encoding phase of WM processing, because greater age-sensitive performance differences were reported when distractors appeared in the encoding phase than in the maintenance phase [4]. Age differences in behavioral performance were examined with response accuracy and latency, whereas neural differences between older and young adults were examined with P2, N2 and LPP amplitudes.

Based on previous research, we hypothesized that older adults would be less accurate and slower than young adults on the 2-back task, and they would have larger P2 and LPP, but smaller N2 amplitudes, than young adults [26, 27, 31, 32]. However, because there have been mixed findings regarding the effects of age-by-valence interactions on WM performance, and there has been no previous ERP study on the time course in resisting emotional distractors within WM among older adults, no specific hypothesis was made regarding the effect of the interaction between age and emotional valence on resistance to distractors, either at the behavioral or neural level.

## Methods

### Participants

The required sample size was estimated with power analysis using G*power 3.1 [40]. Two previous studies exploring emotional WM performance have yielded an average effect size f ~ 0.33 [19, 20]. The power analysis indicated that at least 9 participants in each group would be required to achieve 80% power to detect an effect size of this magnitude. To achieve a sample size that is comparable with previous studies and considering possible drop-out, we aimed to have 30 participants in each age group. Thirty-three older adults (ages over 60 years old) were recruited from the community via posters and word of mouth, and thirty-eight young adults were recruited from the university via online advertisements. One older adult and three young adults were excluded from the analyses due to technical problems during the ERP recording; two older adults ended their participation after the practice session, and voluntarily explained that they found the task boring. Thus, the final sample consisted of 30 older adults (ages 60–74, 14 female) and 35 young adults (ages 19–26, 18 female). By self-report, all participants were right-handed, with normal or corrected-to-normal vision, and no history or current diagnosis of psychological disorders or severe brain damage. The Mini-Mental State Examination (MMSE) [41] was administered to older adults and all of them scored above the cutoff point of 23 (*M* = 27.13, *SD* = 1.25), indicating no cognitive impairment.

Confounding factors that might influence the age-by-valence interactions on WM were also considered and checked as possible control variables. Because the materials included pictures of emotional facial expressions, we administered the Center for Epidemiological Studies Depression Scale (CES-D) [42] and the trait subscale of the State-Trait Anxiety Inventory (STAI-T) [43, 44] to control for the potential confounding effects of emotional distress. In addition, the digit-symbol test and the vocabulary test from the Chinese revision of the Wechsler Adult Intelligence Scale (WAIS-RC) were administered to estimate and account for age differences in processing speed and crystalized intelligence [45].

The study was approved by the research ethics committee of Central China Normal University, and written informed consent was obtained from all participants. The study took roughly 100 min to complete, and participants were paid 80 RMB to compensate them for their time and effort.

### Materials

Emotional faces were selected from the Chinese Facial Affective Picture System (CFAPS) [46], with 28 happy, 28 angry, and 28 neutral facial expressions (14 male pictures and 14 female pictures for each emotion category). The CFAPS database is accessible to approved researchers and institutes. Eight of each facial expression were used as stimuli in the practice block and twenty in the formal experiment. All the emotional faces were black-and-white photographs (7.9 × 6.8°). Based on the CFAPS norms, ratings of the emotional arousal produced by the three kinds of emotional expression were equivalent (*M* ± *SD* for happy: 5.67 ± 0.91; angry: 6.15 ± 1.1; neutral: 5.75 ± 0.2), *F* (2, 81) = 2.626, *p* = 0.078.

### Emotional 2-back task

The emotional 2-back task (adapted from Zhang et al. [47]) was programmed with E-prime 2.0 software. Stimuli were presented on a 23.8-inch LED monitor with a refresh rate of 60 Hz and a resolution of 1920 * 1080 Hz. Participants were seated in a comfortable chair in front of the computer screen, at a viewing distance of 60 cm.

**Fig. 1:**
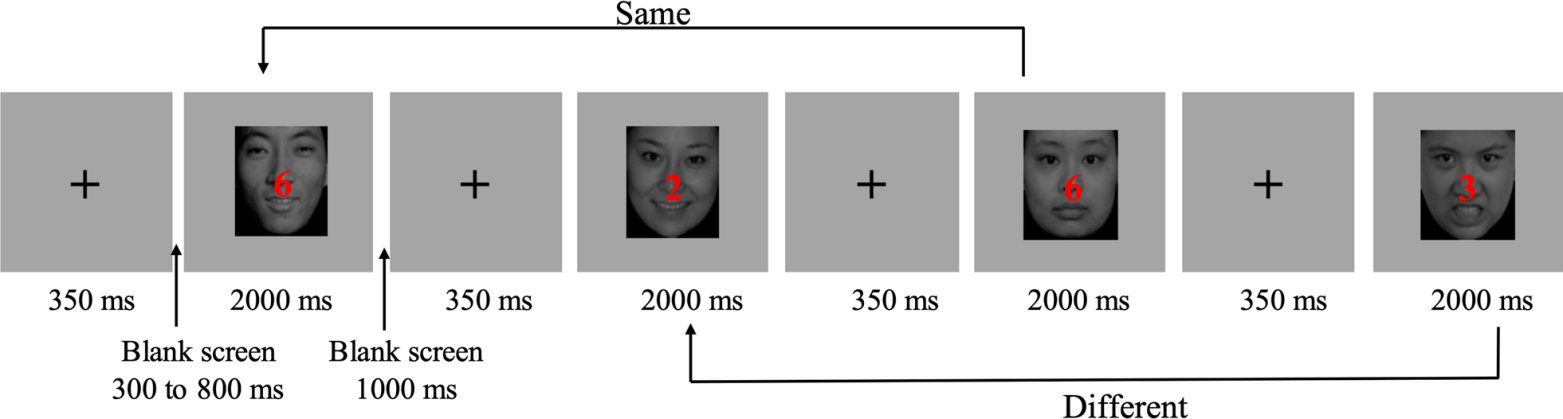
Example of the 2-back task. Participants were instructed to indicate whether the current digit was the same as the one at trial *n* − 2

All stimuli were presented on a gray background (RGB: 192, 192, 192). First, a fixation (0.3 × 0.3°) appeared in the center of the screen for 350 ms, followed by a blank screen for 300 to 800 ms. A digit superimposed on a face was then displayed for 2000 ms, followed by a 1000 ms blank. The digit was randomly selected from the numbers 1 to 9 and was presented in red font. The font size of the digit stimuli was 34 (visual angle of 1.2°). Participants were instructed to indicate whether the current digit was the same as the one at trial *n* − 2 by pressing “F” or “J” with their right or left index finger, respectively. The assignment of keys to indicate “same” or “different” was counterbalanced across participants (Fig. 1).

There was a practice block of 24 trials. The formal experiment had two blocks, each with 60 trials. Half of the trials were “same” trials and half were “different” trials. Each block comprised 20 happy, 20 neutral and 20 negative faces, presented in a pseudorandom sequence. Each face appeared twice, once within each experimental block. The formal experiment did not start until participants achieved an accuracy equal to or higher than 60% during the practice session.

### Electroencephalography (EEG) recording and analysis

The EEG signals were recorded with a 64-channel Ag/AgCl EasyCap™ (Brain Products Gmbh, Germany). The horizontal electrooculogram (EOG) was recorded as the voltage between electrodes placed lateral to the external canthi, and was used to measure horizontal eye movements. The vertical EOG was recorded from an electrode beneath the right eye and was used to detect blinks and vertical eye movements. The reference electrode was set at FCz during recording. The ground point (GND) was set at the midpoint of FPz and Fz. Electroencephalography data were collected with electrode impedances kept below 5 kΩ. The data were digitized with a sampling frequency of 500 Hz and a bandpass filter of 0.05–100 Hz.

Offline analyses were performed using the EEGLAB [48], an open-source Matlab package. The scalp EEG signals were referenced to the average of the left and right mastoids, with a non‐causal Butterworth low‐pass filter of 30 Hz. The recorded EEG data were segmented beginning 200 ms prior to the onset of the stimulus until 1000 ms after the onset. All epochs were baseline-corrected with respect to the mean voltage over the 200 ms preceding the onset of the stimulus. Artifact correction was performed using independent component analysis (ICA), and components corresponding to horizontal and vertical eye movements were removed (typically 2–3 components per participant). Then, epochs containing artifacts exceeding ± 100 µV were removed. Finally, after removing trials with incorrect responses, older adults had about 29 ERP trials under each valence condition, and young adults had about 34 ERP trials under each valence condition.

The effects of positive, negative, and neutral distractors on WM processing were measured by examining three ERP components (P2, N2, and LPP) [49]. The time window of each ERP component was determined based on visual inspection of the separate wave-forms of older and young adults, and based on the time window used in previous studies [27, 31]. More specifically, mean P2 amplitude was calculated by averaging amplitude values at five central-frontal sites: Cz, FC1, FC2, FCz, and Fz, with the time window of 160–220 ms (older adults) or 150–210 ms (young adults). Given the extensive evidence that the N2 is most prominent at midline electrodes [50, 51], and following visual inspection of the scalp distribution map, mean N2 amplitude was calculated by averaging amplitude values at three central-frontal midline sites: Fz, Cz, and FCz, within the time window of 240–310 ms (older adults) or 210–280 ms (young adults). Mean LPP amplitude was calculated by averaging amplitude values at four parietal sites: Cz, Pz, CP1, and CP2 [31], within the time window of 350–600 ms for both older and young adults. Amplitudes of ERP components at each electrode of interest are reported in Additional file 1, for each age group, respectively.

### Statistical analyses

Data were analyzed with SPSS 27.0. Trials with RT less than 100 ms were discarded as deviant [52, 53], and then only data from trials with correct responses were included for further analyses. All participants had RT and accuracy scores that were within three standard deviations of the group mean. Behavioral data (accuracy, RT) and ERP data (P2, N2 and LPP amplitudes) were submitted to five 2 (Age: older adults, young adults) × 3 (Valence: positive, negative, neutral) repeated-measures analyses of covariance (ANCOVAs), with age as the between-subjects factor, and valence as the within-subjects factor. Covariates selection discussed in greater detail below. When the result of an ANCOVA was significant, post hoc testing was conducted to interpret main effects and interactions. Bonferroni correction was used and corrected *p*-values were reported to control for the increased probability of statistically significant results due to multiple comparisons.

## Results

### Demographic information

Characteristics of participants in each age group are summarized in Table 1. Older adults received significantly less education than young adults, *t*(40.424) = 2.963, *p* = 0.005, *d* = 0.77. Young adults scored higher than older adults on the CES-D, *t*(51.796) = 2.464, *p* = 0.017, *d* = 0.59, and on the STAI-T, *t*(63) = 4.858*, p* < 0.001, *d* = 1.21. Young adults performed better than older adults on the digit-symbol test, *t*(63) = 11.952, *p* < 0.001, *d* = 2.97. Older and young adults did not differ in their performance on the vocabulary test, *t*(63) = 0.243, *p* = 0.809, *d* = 0.06. Therefore, years of education, CES-D score, STAI-T score and digit-symbol test score were included in further analyses as covariates to adjust for possible confounds. All covariates were centered across all participants, with the exception of the performance score on the digit-symbol test, which was centered within each age group [54], as previous research has suggested slower processing speed in older adults compared to young adults.

**Table 1:** Participant characteristics, by age group

	Older adults (*n* = 30)		Young adults (*n* = 35)	
	*M*	*SD*	*M*	*SD*
Age	65.5	4.04	20.77	1.61
Years of education	12.4	2.7	14	1.31
CES-D	8.87	4.7	13.29	9.32
STAI-T	29.13	6.79	37.63	7.22
Digit symbol test	41.1	10.96	73.11	10.59
Vocabulary test	57.2	7.93	57.51	8.99

*CES-D* the Center for Epidemiological Studies Depression Scale; *STAI-T* Trait subscale of the State-Trait Anxiety Inventory. The digit symbol test and the vocabulary test are from the Chinese revision of the Wechsler Adult Intelligence Scale (WAIS-RC)

### Behavioral results

#### Accuracy

**Fig. 2:**
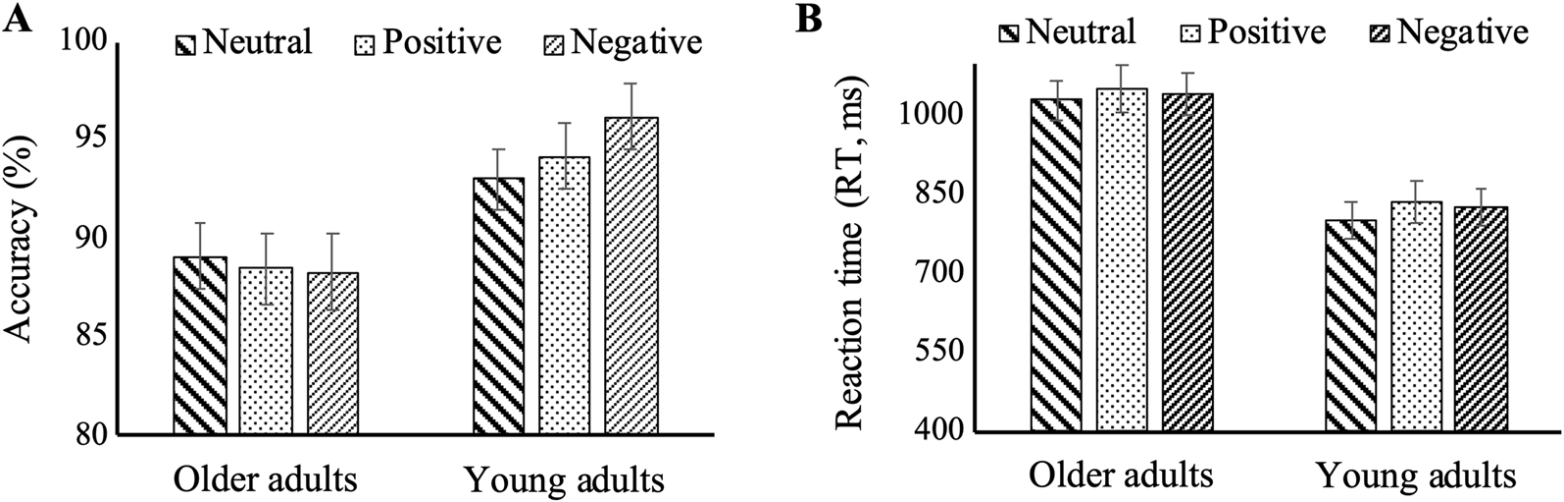
Covariate-adjusted means of response accuracy (**A**) and reaction time (**B**) across three conditions of emotional distractors in older and young adults. Error bars represent the standard error

Behavioral data are presented in Table 2. The 2 (Age: older adults, young adults) × 3 (Valence: positive, negative, neutral) mixed ANCOVA showed a main effect of Age, *F*(1, 59) = 5.288, *p* = 0.025, ${\eta }_{P}^{2}$ = 0.082, where young participants achieved higher accuracy than older adults. The main effect of Valence was not significant, *F*(1.74, 118) = 2.254, *p* = 0.117, ${\eta }_{P}^{2}$ = 0.037. The Age × Valence interaction was significant, *F*(2, 118) = 3.42, *p* = 0.036, ${\eta }_{P}^{2}$ = 0.055. Post hoc analysis showed that within the group of young adults, the accuracy was higher in condition with negative distractors than in conditions with neutral or positive distractors (negative vs. neutral: *p* = 0.004, *d* = 1.69; negative vs. positive: *p* = 0.021, *d* = 1.03), but there was no difference in accuracy between the neutral and positive distractors (*p* = 0.887, *d* = 0.65). However, within the group of older adults, pairwise comparisons between accuracy in the negative, neutral and positive valences conditions showed no effect of valence on accuracy (all *p*s > 0.05) (Fig. 2A). In between-group comparisons, young adults performed better than older adults in the condition with positive distractors (*p* = 0.044, *d* = 3.26) and in the condition with negative distractors (*p* = 0.007, *d* = 4.40). There was no significant difference between young and older adults in the condition with neutral distractors (*p* = 0.117, *d* = 2.44). Regarding covariates, the effect of digit-symbol test score on accuracy was significant, *F*(1, 59) = 5.167, *p* = 0.027, ${\eta }_{P}^{2}$ = 0.081, the faster processing speed, the more accurate response. No other significant effects of covariates on accuracy were found.

**Table 2:** Behavioral and ERP data on the 2-back task in which a digit was superimposed on facial expressions of different emotional valence, by age group

	Older adults	Young adults
	*M* ± *SD*	*M* ± *SD*
**Response accuracy (%)**		
Condition with neutral distractors	88.55 ± 11.00	93.48 ± 4.19
Condition with positive distractors	87.63 ± 13.08	94.89 ± 4.08
Condition with negative distractors	88.03 ± 13.15	96.48 ± 2.99
**Response latency (ms)**		
Condition with neutral distractors	1019.39 ± 180.78	808.44 ± 176.95
Condition with positive distractors	1046.68 ± 222.04	841.07 ± 196.74
Condition with negative distractors	1027.24 ± 190.69	839.11 ± 188.74
**P2 amplitude (μv)**		
Condition with neutral distractors	10.86 ± 5.48	6.69 ± 5.18
Condition with positive distractors	10.81 ± 5.29	6.95 ± 5.76
Condition with negative distractors	11.69 ± 4.96	6.84 ± 5.82
**N2 amplitude (μv)**		
Condition with neutral distractors	3.70 ± 5.34	2.53 ± 5.43
Condition with positive distractors	2.99 ± 4.77	2.12 ± 5.08
Condition with negative distractors	4.46 ± 4.45	3.36 ± 5.90
**LPP amplitude (μv)**		
Condition with neutral distractors	2.17 ± 5.13	3.81 ± 4.70
Condition with positive distractors	1.67 ± 5.43	3.48 ± 4.64
Condition with negative distractors	2.62 ± 5.22	4.21 ± 4.86

#### RT

The 2 (Age: older adults, young adults) × 3 (Valence: positive, negative, neutral) mixed ANCOVA showed a main effect of Age, *F*(1, 59) = 14.46, *p* < 0.001, ${\eta }_{P}^{2}$ = 0.197. Older adults were slower than young adults, indicating lower processing efficiency. The main effect of Valence was also significant, *F*(1.573, 118) = 4.149, *p* = 0.027, ${\eta }_{P}^{2}$= 0.066. Compared to neutral distractors, positive and negative distractors elicited slower responses (positive vs. neutral: *p* = 0.023, *d* = 2.97; negative vs. neutral: *p* = 0.037, *d* = 2.85), but there was no difference in RT between positive and negative distractors, *p* > 0.99, *d* = 0.87. The Age × Valence interaction on RT was not significant, *F*(2, 118) = 0.271, *p* = 0.763, ${\eta }_{P}^{2}$ = 0.005 (Fig. 2B). No significant effects of covariates on RT were found.

### ERP results

**Fig. 3:**
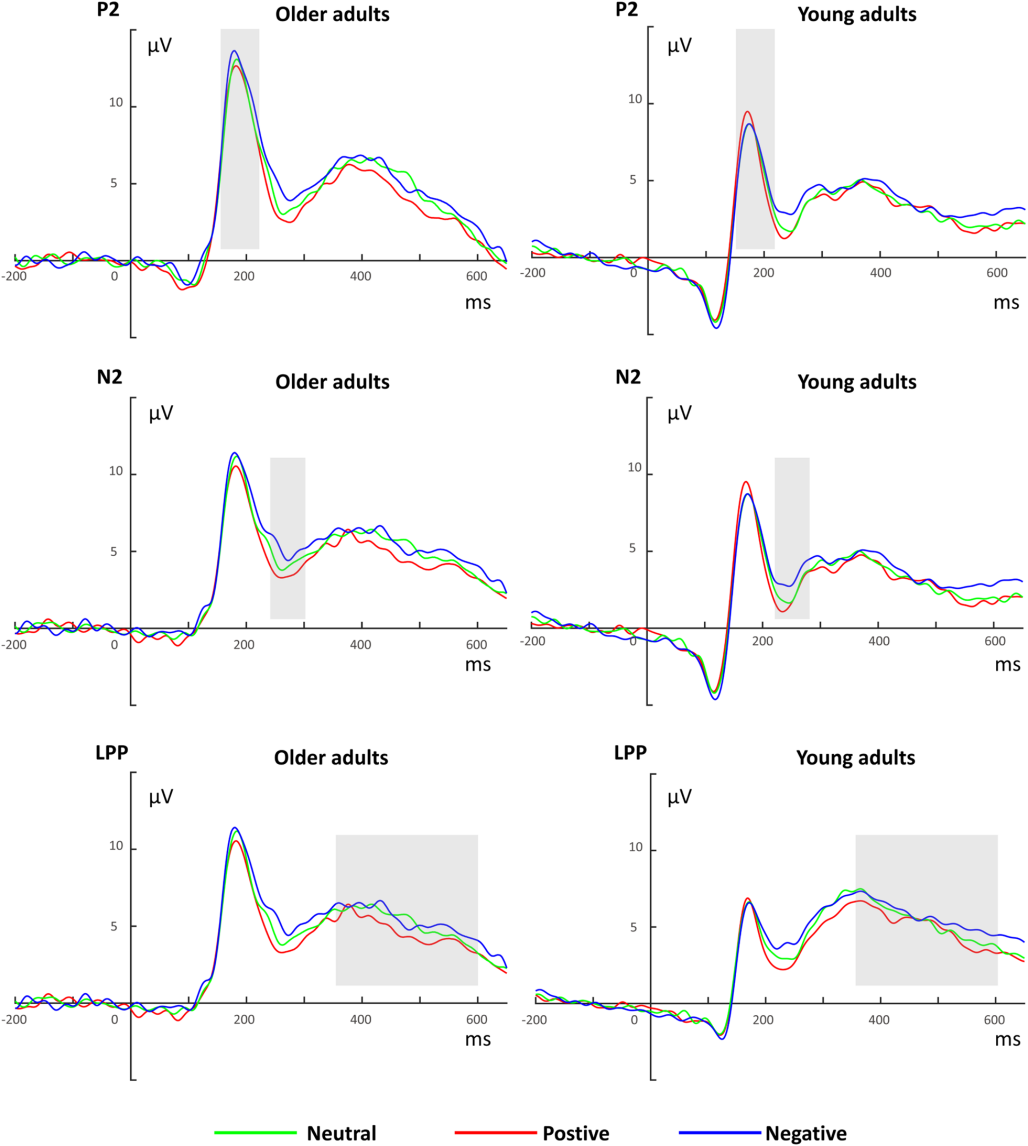
Grand-average ERP waveforms across three conditions of emotional distractors in the *n*-back task in older and young adults. The P2 amplitude was calculated as the average amplitude at Cz, FC1, FC2, FCz, and Fz; the N2 amplitude was calculated as the average amplitude at Fz, Cz and FCz; the LPP amplitude was calculated as the average amplitude at Cz, Pz, CP1 and CP2. Dotted rectangles represent the time windows used for the analysis of each ERP component

Three 2 (Age: older adults, young adults) × 3 (Valence: positive, negative, neutral) mixed ANCOVAs were conducted on P2, N2 and LPP amplitudes (Table 2, Fig. 3). For P2 amplitude, there was a significant main effect of Age, *F*(1, 59) = 6.665, *p* = 0.012, ${\eta }_{P}^{2}$ = 0.101, with older adults showing higher P2 amplitude than young adults. The main effect of Valence, and the two-way interaction between Age and Valence, were not significant, *F*(2, 118) = 1.52, *p* = 0.223, ${\eta }_{P}^{2}$ = 0.025; *F* (2, 118) = 1.068, *p* = 0.347, ${\eta }_{P}^{2}$ = 0.018. One of the covariates, the CES-D score, had a significant main effect on P2, *F*(1, 59) = 4.272, *p* = 0.043, ${\eta }_{P}^{2}$ = 0.068, the more severe symptoms of depression, the smaller P2 amplitude. No other covariate had a significant effect on P2 amplitude.

For N2 amplitude, there was a significant main effect of Valence, *F*(2, 118) = 8.17, *p* < 0.001, ${\eta }_{P}^{2}$ = 0.122. Negative distractors elicited a smaller N2 amplitude than positive distractors (*p* < 0.001, *d* = 4.89), but there was no difference between negative and neutral distractors (*p* = 0.085, *d* = 2.27), or between neutral and positive distractors (*p* = 0.427, *d* = 1.50). The main effect of Age, and the two-way interaction between Age and Valence, were not significant, *F*(1, 59) = 1.587, *p* = 0.213, ${\eta }_{P}^{2}$ = 0.026; *F*(2, 118) = 0.299, *p* = 0.742, ${\eta }_{P}^{2}$ = 0.005. No significant effects of covariates were found.

For LPP amplitude, there was no significant main effect of Age, *F*(1, 59) = 3.156, *p* = 0.081, ${\eta }_{P}^{2}$ = 0.051, main effect of Valence, *F*(2, 118) = 2.676, *p* = 0.073, ${\eta }_{P}^{2}$ = 0.043, or two-way interaction effect, *F*(2, 118) = 0.561, *p* = 0.572, ${\eta }_{P}^{2}$ = 0.009. The covariate CES-D score had a significant main effect on LPP, *F*(1, 59) = 5.888, *p* = 0.018, ${\eta }_{P}^{2}$ = 0.091, the more severe symptoms of depression, the smaller LPP amplitude. No other significant effects of covariates were found.

### Exploratory correlation analysis between WM performance and ERP measures

As the ERP analysis revealed a significant age difference on P2 amplitude, independent of the valence of distractors, we conducted an exploratory analysis of correlations between task performance (accuracy, RT) and P2 amplitude within each age group. A significant correlation between accuracy and P2 amplitude was found within the group of older adults, *r* =  − 0.525, *p* = 0.006, but not within the group of young adults, *r* = 0.234, *p* = 0.204. Thus, greater P2 amplitudes were related to poorer WM performance in older adults. There was no significant correlation between RT and P2 amplitude, either for older adults, *r* = 0.039, *p* = 0.851, or for younger adults, *r* =  − 0.131, *p* = 0.482.

## Discussion

Prior work showed that older adults performed worse than young adults in resisting distractors in WM tasks. However, there is little research on how emotional distractors impact WM function in older adults, or on the neural correlates of resistance to emotional distractors. The present study used behavioral and ERP measures to investigate how emotional distractors impact WM processing in older and young adults. As expected, behavioral results showed that older adults performed more poorly and were slower in resisting distractors than young adults. In addition, an age-by-valence interaction was found for response accuracy, in young adults, negative distractors created a smaller interference effect than positive or neutral distractors. By contrast, there was no evidence of an effect of emotional valence on older adults' resistance to distractors. Together, these behavioral results of decreases in negativity bias with aging provide evidence of age-related positivity effect in WM. The ERP analysis revealed age differences on P2 amplitude but not on N2 or LPP, with greater P2 in older adults than young adults. In both age groups, there was a smaller N2 in the condition with negative distractors than in the condition with positive distractors. The inconsistency between the behavioral and neural results raises the possibility that there are multiple mechanisms underlying the positivity effect in WM.

At the behavioral level, an age-related positivity effect in WM was found, evidenced by the effect of an age-by-valence interaction effect on response accuracy. More specifically, the finding suggests age-related reductions in negativity bias. Similar age-related reductions were observed when the emotional contents were relevant to the task demand [9, 19]. This pattern is also in line with the theoretical predictions of SST, further confirming the fading in negativity bias from youth to old age [6]. However, because measures of general cognitive function were not included, the present research did not enable a direct test of the relation between cognitive abilities and the positivity effect. Therefore, we could not compare the explanatory value of the second-generation theoretical models. Nevertheless, a recent study examining age-related positivity effects in visual attention and episodic memory found no evidence of associations between cognitive capabilities and positivity effects [55]. Further clarification of the role of cognitive functions in shaping positivity effects in WM could help with theoretical integration.

In young adults, negative distractors facilitated WM performance more than positive and neutral distractors did. This finding is consistent with the literature, demonstrating negativity bias in young people [9, 10, 56]. Likewise, a previous study showed that low-arousal negative distractors enhanced attentional performance compared with positive and neutral distractors [57]. By contrast, older adults' WM performance was similar in response to the positive, negative, and neutral distractors. These results are consistent with previous findings showing that WM performance in older adults was not affected by the emotional valence of distractors [18, 24, 58]. For example, in Madill and Murray's study [18], the interfering effect of emotional distractors on a digit identification task was examined with the flanker task. Older adults responded more slowly than young adults, but this effect was not modulated by valence of distracting emotional images. The 2-back task is cognitively demanding, especially for older adults, as it requires continuous monitoring, updating, and manipulation of WM representations [59]. To manage these task demands, older adults might prioritize task goals rather than emotional goals, and use explicit strategies to control interference from distraction, for example by selectively focusing attention on digits that are goal-relevant [18, 60, 61]. This selectivity likely contributes to the attenuated effect of valence on WM performance in older adults. Furthermore, we note that some other studies reported that negative distractors elicited greater intrusion than neutral distractors on the WM performance of older adults [22, 23, 62], which raises questions about the reliability and robustness of the age-related positivity effect in WM.

Age-related alterations in the neurophysiological processing of WM were found only on P2 amplitude, with older adults having a more positive P2 amplitude than young adults, independent of emotional valence. This pattern is consistent with the findings of previous studies that used a basic *n*-back task with numerical stimuli and found greater P2 in older adults [26, 27]. The frontal P2 ERP component has been linked to post-perceptual selective attention and early WM encoding. Therefore, the larger P2 might reflect difficulties in older adults in comparing the incoming stimulus with the mental representation of the previous stimulus during the early (encoding) phase of WM processing. Similarly, a previous study showed that depressed participants, who were characterized by deficits in WM, had greater P2 amplitude than nondepressed participants across neutral and emotional WM conditions [31]. It might also be possible that the greater P2 in older adults reflects effort in attempting to compensate for age-related declines in WM function. However, in the current study there was a negative association between P2 amplitude and accuracy, contradicting the compensation account. By contrast, there were no significant associations between P2 amplitude and response latency, and thus the longer latency in older adults might reflect a general slowing that does not relate to specific WM operations. This further demonstrates that response accuracy and latency represent two independent indicators of WM function [63].

A smaller N2 was found for negative distractors compared to positive distractors in both age groups. The N2 amplitude reflects attentional conflict processing, which is more negative for conflict trials compared to non-conflict trials [33, 64]. Previous studies of young adults also found smaller interference from negative distractors than positive distractors [58], and N2 was smaller for angry faces than happy faces [65]. In the current study, the behavioral results showed that both negative and positive distractors were associated with slower responses than neutral distractors, suggesting that both negative and positive distractors are emotionally salient and are prioritized in capturing attention. However, negative stimuli have adaptive value in evolution, and people usually attend to and process negative information relatively automatically. Whereas distractor resistance and target processing compete for the limited cognitive resources of WM, when less cognitive control is required in resolving conflict from negative emotional distractors, the target is processed more efficiently [66], reflected in the smaller N2. Regarding the effect of age on N2, contrary to our hypothesis, no significant age difference was observed. This null result is inconsistent with previous studies that reported smaller N2 amplitudes in older adults than young adults in a numeric *n*-back task [26, 27], but no distractors were involved in these studies. In addition, one study found that when older adults were classified as having high or low WM, no difference in N2 between either older adult group and their young adult counterparts [26], suggesting a confounding effect of individual difference in WM task performance on N2 amplitude.

Age was not found to have a significant effect on the LPP amplitude, which was not consistent with our predictions. Studies in emotional memory have reported evidence for the positivity effect on LPP amplitude, especially on the later portion of LPP [67, 68]. The LPP in this study may index the later maintenance process during WM updating. While early WM processing is relatively effortful, late WM processing is relatively automatic [69] and this explains why age-related impairment appears only during the early process of WM [35, 70]. In addition, there was no difference on LPP across the three valence conditions. Another study also reported similar LPP amplitudes across the three emotional valence conditions in a 2-back task with emotional words [31]. These findings, however, are not completely consistent with previous studies. For example, multiple studies have found a greater LPP amplitude for emotional distractors than neutral distractors [35, 36, 71]. A significant source of the inconsistency might be the phase of WM in which the distractor was displayed. Whereas distractors were displayed during WM maintenance in these previous studies, distractors were displayed during WM encoding in the present study. Studies exploring the effects of distractors on WM performance have demonstrated that people are affected differently by distractors displayed at encoding or during the maintenance period [72, 73]. In addition, the recent literature suggests that the magnitude of the LPP component might be a robust measure of WM load, with smaller LPP under higher WM load [74–76]. As such, it would be interesting to further investigate how the interaction between WM load and the emotional valence of distractors may impact the time course of distractor resistance across different age groups.

In this study we combined behavioral and neurophysiological measures to investigate how emotional distractors impact WM performance in older and young adults. Discrepancies between behavioral and ERPs results challenge conclusions made about age-related positivity effects in WM. Yet, it is noteworthy that research has suggested that there are multiple sources of age differences in interference control [77, 78], and behavioral and neural measures may elucidate different facets of age-related changes in emotional WM processing [79]. The current findings have important implications for future studies of how emotion distractors impact WM performance in older adults. For example, the functional imaging technique has been used to determine the neural mechanism underlying emotional distractor resistance during WM in young adults [80, 81]. By combining the neuroimaging method with ERP measurement, future research could examine how emotional distractors alter brain activation patterns over a longer time period, and whether neural reorganization and compensation in aging occur [82]. Furthermore, recent research extended the SST and supported a dual-process account of the positivity effect [83, 84]. This model raises intriguing questions about how aging might have a different effect on the automatic and effortful control of interference from emotional distractors. Further research should therefore aim to identify the factors that modulate the magnitude and direction of age differences in resisting emotional distractors in WM [85].

### Limitations

The present study has some limitations. First, our participants were not matched on level of depression or anxiety. Although these variables were included as covariates, further investigation is required in groups of participants with similar levels of emotional distress. Secondly, while we selected faces from the CAPS based on normative ratings, the valence and arousal ratings of facial expressions might differ between age groups. For example, older adults were found in other research to have greater difficulty identifying negative emotions, and they rated emotional faces as more arousing than their young counterparts did [86]. Thirdly, a recent study revealed age-related differences in the sequential modulation of effects of emotion on cognitive performance [87], and the present findings might be confounded by the sequence of emotional faces.

## Conclusions

This work investigated the behavioral and neurophysiological effects of emotional distractors on WM processing in older and young adults. The behavioral results provided support for the age-related positivity effect in WM, which was evidenced in the current study as a reduction in negativity bias with aging. The neurophysiological results showed that compared to young adults, older adults displayed larger P2 amplitude in the early WM encoding phase; however, this age difference did not vary across emotional valences. This discrepancy between the behavioral and neural findings suggests that there may be multiple mechanisms underlying the positivity effect in WM. The nature and source of these intriguing age differences should be further examined, which could help to determine which theoretical approach provides the best explanation for this phenomenon.

## Supplementary Information

**Additional file 1**. Amplitudes of ERP components at each electrode of interest, for each age group, respectively.

## Data Availability

The datasets used and analyzed in the current study are available from the corresponding author on reasonable request.

## Abbreviations

WM: Working memory
LPP: Later positive potential
RT: Reaction time
